# Exploring the Dynamics of Athletes’ Enjoyment and Self-Determined Motivation, and of the Motivational Climate in Youth Football: A Longitudinal Perspective

**DOI:** 10.1177/00315125231222152

**Published:** 2023-12-14

**Authors:** Filipe Rodrigues, Diogo Monteiro, Rui Matos, Miguel Jacinto, Raúl Antunes, Nuno Amaro

**Affiliations:** 1 70867 ESECS - Polytechnic of Leiria, Leiria, Portugal; 2 Research Center in Sport, Health, and Human Development (CIDESD), Vila Real, Portugal

**Keywords:** achievement goal theory, self-determination theory, motivation, football, youth

## Abstract

We aimed to explore a short period longitudinal interplay between athletes’ enjoyment and their self-determined motivation and motivational climate in youth football. We recruited 109 youth football athletes (79 males, 30 females) through a convenient sampling method. We included individuals within the 12–17-year-old age range, with a mean age of 14.31 (*SD* = 1.46) years. To examine these proposed associations, we performed hierarchical multiple regression analyses and found that enjoyment at pre-season assessment (T1) and self-determined motivation and a task-involving sport climate at mid-season (T2) were significant predictors of mid-season enjoyment (at T2). However, self-determined motivation and task-involving climate at T1 did not significantly contribute to the model. These findings emphasize the importance of initial enjoyment and an evolved self-determined motivation, and task-involving climate in understanding later enjoyment in sport. Coaches, practitioners, and policymakers should prioritize strategies that enhance intrinsic motivation, provide opportunities for autonomy, and cultivate a supportive and growth-oriented environment.

## Introduction

Football, renowned as one of the world’s most popular sports, has shown an unparalleled ability to captivate the minds and hearts of countless players and fans globally. Its dynamic action, skilled performances, and an associated unwavering competitive spirit make each match an electrifying spectacle (Dvorak et al., 2004). Nevertheless, the emotional significance of football transcends its physical demands and strategic intricacies. Whether as a player striving to score the winning goal or as a fervent fan passionately supporting their beloved team, football enthusiasts experience an exhilaration that can be immensely gratifying. The sheer thrill of participating in football, the satisfaction derived from executing skillful maneuvers, and the camaraderie fostered among teammates collectively interact with coaching influences to contribute to overarching enjoyment (Cresswell et al., 2019; Vaughan et al., 2022). In the context of youth football, there can be an even more crucial impact of self-determined motivation on enjoyment. Those young players who are intrinsically motivated find joy in the game itself, rather than in external factors like rewards or pressure, and they are more likely to have a positive and fulfilling experience (Farhani et al., 2022; Kavussanu, 2006).

### Key Motivational Determinants of Enjoyment

In youth football, the coach’s role becomes paramount. Coaches’ interpersonal behaviors and approaches heavily influence the development of self-determined motivation in young players. Coaches who provide support, encouragement, and opportunities for autonomy can enhance players' intrinsic motivation (Rodrigues, et al., 2020a). Achievement goal theory, specifically the task and ego-involving motivational climate, aligns with this development of self-determined motivation and seeks to explain how individuals approach and strive for achievement in different contexts (Harwood et al., 2008). Developed by Nicholls (1984) and explored by Roberts and colleagues (Roberts et al., 1998) in the sport context, achievement goal theory focuses on the goals individuals pursue and the underlying motivations that drive their behavior. There are two primary motivational climates that players may perceive: task and ego-involving motivational climates.

A task-involving climate in youth football is characterized by an emphasis on personal improvement, skill development, and effort. For this climate, coaches and adults involved in the sport create an atmosphere that values learning, skill mastery, and individual progress. They provide support, encouragement, and constructive feedback to help athletes develop their abilities and reach their full potential (Alesi et al., 2019; Roberts et al., 1998; Rodrigues, et al., 2020b). On the other hand, an ego-involving motivational climate places a strong emphasis on winning, social comparison, and outperforming others. This climate can lead to more prevalent extrinsic motivation, wherein players are driven by rewards, recognition, and social status. Hence, within the football context, this theory suggests that the type of player perceived motivational climate can significantly impact players’ motivation, behavior, and performance (Amaro et al., 2023).

Prior research suggests that fostering a task-involving motivational climate in youth football contributes to the development of self-determined motivation (Fabra et al., 2023; Monteiro, et al., 2018). Self-determination theory (Ryan & Deci, 2017) provides valuable insights into motivation and how it relates to different motivational states, including a task-involving motivational climate. Players who perceive their coaches as supportive people who value their effort, personal growth, and cooperation, are more likely to experience self-determined motivation (Ntoumanis, 2001). Self-determined motivation refers to the motivation that arises from within an individual. Intrinsic motivation, integrated regulation, and identified regulation are all components of self-determined motivation within the framework of self-determination theory. They represent different levels of internalization and personal endorsement of an activity (Ryan & Deci, 2017). Intrinsic motivation is the highest level of self-determined motivation. It refers to engagement in an activity purely for its inherent interest or the personal satisfaction one derives from the activity itself. Integrated regulation involves engaging in an activity because it aligns with one’s values, beliefs, or personal identity. Identified regulation occurs when individuals engage in an activity because they personally value or recognize its importance.

When athletes are motivated in a self-determined way, they engage in an activity because they genuinely find it meaningful, satisfying, or aligned with their values and personal goals. Research suggests that self-determined motivation is associated with numerous positive outcomes, such as increased persistence, better performance, higher well-being, and more sustainable engagement in activities (Calvo et al., 2010; Pelletier et al., 2001; Sarrazin et al., 2002). In addition, self-determined motivation is positively associated with enjoyment in various activities, including youth football, as it reflects enjoyment and fulfillment. However, it is important to recognize that the level of self-determined motivation and its corresponding enjoyment can fluctuate over time, influenced by factors such as coaching practices and social support within the sport (Haas et al., 2021; Rodrigues, et al., 2020a).

### Current Study

Most studies examining the relationship between enjoyment and key motivational determinants in youth football have utilized a cross-sectional research design in which variables can be significantly associated, but interpretations of possible causal influences between variables can only be suspected or assumed (Monteiro, et al., 2018; Rodrigues et al., 2023). Cross-sectional studies provide a snapshot of data at a specific point, offering insights into associations without capturing the dynamic or changing nature of enjoyment and motivation throughout a competitive season. While the associations between enjoyment, self-determined motivation, and motivational climate on football participation have been widely acknowledged, it is essential to examine how these variables change over time (Amaro et al., 2023). Longitudinal studies allow us to capture the dynamic nature of these constructs, unveiling how they evolve as players progress through different stages of their football careers (Ruiz et al., 2019). By understanding these trajectories of enjoyment, self-determined motivation, and the motivational climate, we can develop strategies to promote sustained engagement and well-being in football. Longitudinal studies that follow individuals’ athletic development over an extended period allow for a particularly comprehensive understanding of how enjoyment, self-determined motivation, and the motivational climate fluctuate across different stages of engagement (Fabra et al., 2023). Such insights can guide the development of evidence-based interventions, training programs, and policies that maximize the positive impact of football and cultivate a lifelong love for the game.

In the present article, we aimed to explore the interplay between enjoyment, self-determined motivation, and the motivational climate in youth football from a short-term longitudinal perspective. Drawing upon existing research and empirical evidence, we delved into the factors that influence changes in these variables over time. In this way, we hoped to provide valuable insights for practitioners, coaches, clubs, and policymakers in their efforts to create supportive environments that nurture enjoyment, foster self-determined motivation, and enhance the overall youth football experience. We hypothesized that self-determined motivation would be positively associated with enjoyment. In addition, we expected that athletes’ perceptions of a task-involving climate would be positively correlated with self-determined motivation.

## Method

### Participants

We employed the A-priori Sample Size Calculator for Multiple Regression (Soper, 2022) to determine the minimum required sample size for sufficient statistical power for this study. We set the anticipated effect size (f2) at .15, the desired statistical power level at .8, the number of predictors at 5, and statistical significance at .05. These assumptions led to an estimated minimum required sample size of 91. Considering the necessary precautions for potential outliers and missing data, we collected data for an additional 10% of participants.

We recruited 109 youth football athletes (79 males, 30 females) through a convenient sampling method. We included individuals within the age range of 12–17 years, with a mean age of 14.31 (*SD* = 1.46). All athletes were actively involved in football training at a local club. The reported frequency of practice sessions ranged from 2–4 times per week, with an average practice frequency of 3.24 (*SD* = .54) times per week. The athletes' experiences in football ranged from 1 to 13 years (*M* = 7.07, *SD* = 2.45). By age groups, our participants were: (a) under 12 (*n* = 19), (b) 12–13 (*n* = 35), (c) 14–17 (*n* = 20), and (d) 18–19 (*n* = 35).

### Data Collection Procedures

All data collection procedures adhered to the principles preserved in the Helsinki Declaration (World Medical Association, 2013). Prior to commencing data collection, ethical approval was obtained from the Ethics Committee (PARECER N. ° CE/IPLEIRIA/24/2021). Through a convenience sampling approach, we first contacted a local football academy and sought permission from the director to conduct our study. The study was carried out in partnership with a local football academy that serves both male and female students. Although this academy does not match the intensity of elite professional teenage academies, it maintains a well-organized training routine to ensure continuous development and progress for its participants. Following a positive response, we proceeded to establish communication with the parents or legal guardians of the participating adolescents. During these interactions, a comprehensive presentation was made regarding the nature of the study, ethical considerations, and the data collection procedures. We obtained informed consent from the parents or legal guardians who granted permission for the inclusion of their children in the research investigation. Prior to the distribution of the questionnaires, a detailed explanation of the study’s objectives was provided to all athletes. They were informed of their right to voluntarily withdraw from participation at any given point and were requested to provide their informed assent before proceeding with the questionnaire completion. Rigorous protocols were established to safeguard participant anonymity during the data collection process.

The questionnaires were administered to the participants prior to their training sessions. Data were collected at two distinct time points: (i) two weeks prior to the commencement (i.e., august) of the competitive season (T1) and (ii) at the midpoint (i.e., February) of the season (T2). Participants completed the questionnaires in the absence of their coaches to ensure unbiased responses. They were encouraged to provide honest answers and seek clarification whenever necessary. Furthermore, participants were requested to report their perceptions of their current coach.

### Instruments

We assessed the task-involving motivational climate with the Motivational Climate Sport Youth Scale, specifically the Portuguese version (Monteiro, et al., 2018). This scale consists of eight items that were rated on a five-point Likert scale, ranging from 1 (“Totally Disagree”) to 5 (“Totally Agree”). In this study, the focus was specifically on the task-involving climate subscale items, which exhibited favorable internal consistency (α_T1_ = .71; α_T2_ = .79). These four items were selected as they aligned with the theoretical construct of achievement goal theory, specifically reflecting the task-involving climate (e.g., “The coach encouraged us to learn new skills”). This questionnaire has demonstrated robust psychometric properties, including satisfactory internal consistency, indicating its effectiveness in assessing the intended constructs (Monteiro, et al., 2018).

To assess self-determined forms of motivation, we utilized the Behavioral Regulation Sport Questionnaire, Portuguese version (Monteiro et al., 2019). This questionnaire consists of a total of 24 items, and respondents provided their answers on a seven-point Likert scale ranging from 1 (“Not True for Me at All”) to 7 (“Completely True for Me”). The items are categorized into six factors, each comprising four items, which represent different types of motivation along the motivational continuum. In this study, our focus was on the items related to three subscales: identified regulation (e.g., “Because I recognize the benefits of my sport”), integrated regulation (e.g., “Because it is a part of who I am”), and intrinsic motivation (e.g., “Because I find it enjoyable”). We assessed the internal consistency of these subscales, and these results indicated satisfactory reliability: identified regulation (α_T1_ = .71; α_T2_ = .80), integrated regulation (α_T1_ = .79; α_T2_ = .82), and intrinsic motivation (α_T1_ = .80; α_T2_ = .81) as well as the self-determined motivation (α_T1_ = .70; α_T2_ = .83). This questionnaire has demonstrated robust psychometric properties, including satisfactory internal consistency, indicating its effectiveness in assessing the intended constructs (Monteiro, et al., 2018).

To assess the cognitive aspect of enjoyment during sport practice, we utilized the of the Physical Activity Enjoyment Scale Portuguese version (Monteiro et al., 2017). This scale consists of eight items, and participants rated their responses on a 5-point Likert scale ranging from 1 (“totally disagree”) to 5 (“totally agree”). The items were combined into a single factor that represented the participants’ level of enjoyment (e.g., “I find it pleasurable”), indicating a positive response to the sports practice experience. In our study, this scale demonstrated a high level of internal consistency (α_T1_ = .85; α_T2_ = .91). This survey has exhibited strong psychometric characteristics, including adequate internal consistency, affirming its efficacy in evaluating the targeted constructs (Monteiro et al., 2017).

### Statistical Analysis

Data analyses were performed using IBM SPSS Version 26.0 software (IBM Corp, Armonk, NY). Missing data were handled using the expectation-maximization approach. Descriptive statistics were reported as means and standard deviations and frequency percentages. To assess any deviation from normal distribution, we estimated skewness and kurtosis and divided by their corresponding standard error to obtain z scores. A z score below |1.96| indicated a normal distribution (Wright & Herrington, 2011). Bivariate correlations were examined among variables of interest, with partial correlations conducted while controlling for sex. The significance level was set at *p* ≤ .05 to reject the null hypothesis. Internal consistency was assessed using Cronbach’s alpha, considering coefficients of ≥ .70 as acceptable (Cronbach, 1951).

To examine the proposed associations, we performed hierarchical multiple regression analyses. Prior to regression analysis, we conducted tolerance tests and Variance Inflation Factor (VIF) scores to detect any multicollinearity (Cohen, 1988). Tolerance values above .1 indicated the absence of multicollinearity. The Durbin Watson statistic was calculated to test for autocorrelation, with an acceptable range of 1.50–2.50 (Durbin & Watson, 1950). The dependent variable in this analysis was enjoyment at T2. The stepwise regression procedure was employed, adding variables based on the model assumptions outlined in the introduction section. Model 1 included enjoyment at T1, while Model 2 included self-determined motivation at T2. Model 3 incorporated self-determined motivation at T1, Model 4 included task-involving climate at T2, and finally, Model 5 included task-involving climate at T1. Model comparisons were made using R2, and changes in adjusted R2 were analyzed with a significance level of ≤.05 to reject the null hypothesis.

## Results

Data from 13 participants were not collected at T2, due to their absence during the assessment process. To address these missing data, we performed imputation using the expectation-maximization approach*. Table 1 displays the descriptive statistics and bivariate correlations for the remaining participants. Analyzing these data, we observed no significant changes between the time points for any variable under analysis. This finding was confirmed by paired sample t-tests, which indicated that the differences between scores at T1 and T2 were not statistically significant (*p* > .05). The data distributions for skewness and kurtosis were within acceptable limits, indicating normal distributions. As anticipated, several significant bivariate correlations emerged: (a) Task-involving climate at T1 was positively correlated with both task-involving climate at T2 (r = .22, *p* < .024) and self-determined motivation at T1 (r = .21, *p* < .031); (b) Task-involving climate at T2 was positively correlated with self-determined motivation at T1 (r = .40, *p* < .001) and self-determined motivation T2 (r = .40, *p* < .001); (c) self-determined motivation at T1 was positively correlated with self-determined motivation at T2 (r = .48, *p* < .001) and Enjoyment at T1 (r = .38, *p* < .001); (d) self-determined motivation at T2 was positively correlated with both Enjoyment at T1 (r = .48, *p* < .01) and Enjoyment at T2 (r = .47, *p* < .001); and, (e) enjoyment at T1 was positively correlated with enjoyment at T2 (r = .51, *p* < .001).

**Table 1.** Descriptive Statistics and Correlational Analysis.

Variables	M	SD	S	K	1	2	3	4	5	6
1. Task-involving climate T1	4.45	.41	−.45	−.57	1					
2. Task-involving climate T2	4.43	.42	−.56	.13	.22*	1				
3. Self-determined motivation T1	5.68	.76	−.45	−.25	.21*	.40**	1			
4. Self-determined motivation T2	5.69	.88	−1.02	2.39	.02	.40**	.48**	1		
5. Enjoyment T1	6.10	.68	−.68	−.06	.62**	.38**	.43**	.14	1	
6. Enjoyment T2	6.04	.76	−.44	−.84	.39**	.56**	.36**	.47**	.51**	1

*Notes*: M = mean; SD = standard-deviation; S = skewness; K = kurtosis; below diagonal line = bivariate correlations; *p < .05; **p < .01

Table 2 presents the results of the hierarchical multiple regression analysis. The tolerance values ranged from .61 to 1.00, indicating no significant multicollinearity issues. Similarly, the VIF values ranged from 1.00 to 2.03, further confirming the absence of multicollinearity. To assess autocorrelation, the Durbin–Watson test yielded a score of 1.89, indicating a score close to zero and suggesting minimal autocorrelation. The significance (*p*-value) of each model was examined to determine if it significantly differed from a null hypothesis. Additionally, the R2 value was evaluated to determine the proportion of variance in enjoyment at T2 explained by the model. To identify the variable that contributed the most to the model, the standardized coefficients and significance of the independent variables were examined. In the hierarchical multiple regression analysis, the models were compared as variables were added, focusing on the changes in R2 to assess the incremental contribution of each variable.

**Table 2.** Hierarchical Multiple Regression Analysis.

Model	β	t	R	R2	ΔR2	df	F
Model 1			.26	.25	.26	1, 107	<.001
Enjoyment T1	.51**	6.12					
Model 2			.42	.41	.16	1, 106	<.001
Enjoyment T1	.45**	6.08					
Self-determined motivation T2	.41**	5.45					
Model 3			.42	.41	.00	1, 105	.58
Enjoyment T1	.47**	5.73					
Self-determined motivation T2	.43**	5.04					
Self-determined motivation T1	−.05	−.06					
Model 4			.49	.47	.07	1, 105	<.001
Enjoyment T1	.38**	4.73					
Self-determined motivation T2	.33**	3.98					
Self-determined motivation T1	−.09	−1.02					
Task-involving climate T2	.31**	3.77					
Model 5			.51	.48	.01	1, 103	.08
Enjoyment T1	.29**	2.89					
Self-determined motivation T2	.34**	4.11					
Self-determined motivation T1	−.08	−.94					
Task-involving climate T2	.31**	3.78					
Task-involving climate T1	.15	1.73					

*Notes*: β = standardized coefficients; t = *t* test; R2 = adjusted r square - explained variance; Δ = differences; F = changes in significance; df = degrees of freedom; *p < .05; **p < .01.

The hierarchical multiple regression analysis results are presented in Table 2. In Model 1, the inclusion of Enjoyment at T1 as the sole predictor yielded a significant regression equation (β = .51, t = 6.12, *p* < .001), explaining 26% of the variance on enjoyment at T2 (R2 = .26, *p* < .001). In Model 2, a second predictor was added to enjoyment T1 (β = .45, t = 6.08, *p* < .001): self-determined motivation T2 (β = .41, t = 5.45, *p* < .001). This model showed an improved fit (R2 = .41, ΔR2 = .16, *p* < .001), explaining a total of 42% of the variance in enjoyment at T2. Model 3 included enjoyment at T1, self-determined motivation at T2, and self-determined motivation at T1 as predictors. However, the addition of Self-determined motivation at T1 did not contribute significantly to the model (β = −.05, t = −.06, *p* > .05), and the explained variance remained unchanged (R2 = .41, ΔR2 = .00, *p* = .58). Model 4 introduced task-involving climate at T2 as an additional predictor. The model exhibited a significant improvement (R2 = .47, ΔR2 = .07, *p* < .001), with enjoyment at T1 (β = .38, t = 4.73, *p* < .001), self-determined motivation at T2 (β = .33, t = 3.98, *p* < .001), and task-involving climate at T2 (β = .31, t = 3.77, *p* < .001) contributing significantly. Finally, Model 5 included Task-Involving Climate at T1 in addition to the previous predictors. However, the contribution of Task-Involving Climate at T1 was not statistically significant (β = .15, t = 1.73, *p* = .086), and the explained variance remained unchanged (R2 = .48, ΔR2 = .01, *p* = .08).

These findings suggest that enjoyment at T2, self-determined motivation at T2, and task-involving climate at T2 were significant predictors of enjoyment at T2, while self-determined motivation at T1 and task-Involving Climate at T1 did not significantly contribute to the model.

## Discussion

The present study aimed to examine the interplay between enjoyment, self-determined motivation, and the motivational climate in youth football using a longitudinal perspective. This study built upon existing research and empirical evidence to gain a deeper understanding of the factors influencing these variables and their potential fluctuations over time. By uncovering these dynamics, we sought to provide valuable insights for practitioners, coaches, clubs, and policymakers in creating supportive environments that foster enjoyment, enhance self-determined motivation, and improve the overall football experience.

The results of our hierarchical multiple regression analysis provided valuable insights into the predictors of enjoyment in the context of youth football. These findings suggested that enjoyment at T2 was a significant predictor of future higher levels of enjoyment at T2. This aligns with previous research that highlighted the positive impact of enjoyment on sustained engagement in sports (Calvo et al., 2010; Pelletier et al., 2001; Sarrazin et al., 2002). Theoretical evidence from positive psychology and self-determination theory supports the link between enjoyment and sustained engagement in sports. Positive psychology emphasizes the role of enjoyment, such as enjoyment, in promoting well-being and optimal functioning. In the context of youth football, experiencing enjoyment during the activity is likely to contribute to a sense of happiness, satisfaction, and fulfillment (Calvo et al., 2010). This, in turn, can foster a positive feedback loop, in which individuals continue to engage in the activity because it brings them joy and enhances their overall well-being (Farhani et al., 2022). Numerous studies have examined the relationship between enjoyment at T1 and T2 or participation in various sports contexts. For example, research investigating the determinants of enjoyment in youth football found that higher levels of enjoyment were associated with greater commitment and continued participation in the sport. This suggests that enjoyment plays a pivotal role in sustaining engagement and involvement in youth football.

The inclusion of self-determined motivation at T2 in Model 2 and its improvement in predictive power highlight the crucial role that self-determined motivation plays in shaping enjoyment in the context of youth football. This finding underscores the importance of fostering internalized motivation and personal endorsement of the activity. Individuals who perceive their engagement in football as autonomous are more likely to experience higher levels of enjoyment (Ryan & Deci, 2017). Theoretical evidence from self-determination theory provides a framework for understanding the relationship between self-determined motivation and enjoyment. When individuals feel a sense of autonomy and choice in their engagement in football, it enhances their intrinsic motivation and contributes to a more enjoyable experience (Garcia-Mas et al., 2010; Monteiro, et al., 2018).

The finding that the addition of self-determined motivation at T1 did not contribute significantly to the model, and that the explained variance remained unchanged suggests that self-determined motivation at T1 did not have a significant impact on enjoyment at T2. This finding indicates that an athlete’s current level of self-determined motivation (T2) is a stronger predictor of enjoyment than their past level of enjoyment (T1). Several theoretical and empirical explanations can shed light on why self-determined motivation at T2 predicted enjoyment at T2 while self-determined motivation at T1 did not significantly contribute to the model. Temporal dynamics of motivation could explain the current results, especially since they are different moments of the season (pre-competitive moment vs. competitive moment). Self-determined motivation can vary over time due to changing circumstances, experiences, or personal growth (Ryan & Deci, 2017). The motivational state at T2, which is closer to the time of assessing enjoyment at T2, may be a more accurate reflection of the individual’s current motivational orientation. There appears to be a recency effect in which recent experiences or states have a stronger impact on judgments or predictions compared to earlier experiences or states. In the context of self-determined motivation and enjoyment, individuals may prioritize and weigh their current level of self-determined motivation (T2) most heavily when assessing their enjoyment of the activity at the same time point (T2). This recency effect supports the notion that the temporal proximity of the predictor to the outcome assessment is crucial in understanding its predictive power (Garcia-Mas et al., 2010). These explanations highlight the importance of considering the current motivational state when predicting enjoyment and suggest that recent motivation may be a more salient factor in shaping the experience of enjoyment (Ryan & Ryan, 2023).

The finding that the introduction of a task-involving climate at T2 in Model 4 significantly improved the predictive power of the model suggests that creating an environment that emphasizes personal improvement, skill development, and effort is conducive to fostering enjoyment in youth football. This finding aligns with theoretical perspectives (Kavussanu, 2006). A task-involved climate focuses on personal improvement, skill development, and effort, while an ego-involved goal orientation centers around demonstrating superiority and outperforming others. Research has consistently shown that a task-involving climate, characterized by an emphasis on effort, improvement, and mastery, is associated with positive outcomes such as enjoyment, intrinsic motivation, and persistence in sports participation (Alesi et al., 2019; Pelletier et al., 2001). When youth football players perceive their environment as task-involving, they are more likely to experience enjoyment, due to the focus on personal growth and the positive learning experiences fostered by this climate (Mossman & Cronin, 2019). A study in youth football investigated the impact of the motivational climate on athletes' emotional experiences, including enjoyment (Monteiro, et al., 2018). The results revealed that a task-involving climate was associated with more positive affective experiences, including higher levels of enjoyment. This suggests that creating an environment that emphasizes personal improvement and effort can enhance the enjoyment of young athletes in football.

The fact that Model 5 included task-involving climate at T1 as a predictor but did not find it to be statistically significant in predicting enjoyment at T2 indicates that the perception of the task-involving climate at T2 had a stronger influence on enjoyment compared to the perception at T1. Several factors could contribute to this finding. The lack of statistical significance and unchanged explained variance for task-involving climate at T1 in predicting enjoyment at T2 suggests that the current perception of the task-involving climate (T2) may be more salient and relevant in influencing enjoyment than the past perception (T1). It is possible that the immediate or recent experiences of the task-involving climate have a greater impact on enjoyment than the earlier experiences. This highlights the dynamic nature of motivational factors and suggests that the most recent perceptions are more influential in shaping the subjective experience of enjoyment. It is also plausible that the task-involving climate experienced by the participants in the study underwent changes between T1 and T2. Environmental factors, such as coaching practices, team dynamics, or changes in the overall sports context, might have contributed to the variance in the task-involving climate over time (Cresswell et al., 2019). These changes may have influenced the strength of the relationship between the task-involving climate and enjoyment, making the T2 perception a more relevant predictor of enjoyment. Youth football is a dynamic context, characterized by individual and social development. As players progress and develop in their skills, knowledge, and social interactions, their perceptions of the task-involving climate may change (Ruiz et al., 2019; Ryan & Ryan, 2023). The evolving nature of the developmental process might explain why the task-involving climate at T2 impacted enjoyment more strongly than at T1. The players' evolved understanding of the sport and their evolved needs and expectations may have contributed to this differential influence. While the statistical significance of a task-involving climate at T1 was not observed to predict enjoyment at T2 in this particular study, it is important to consider that statistical significance does not necessarily imply practical significance or real-world impact. The small effect size observed (β = .15) might still have some practical significance or may be relevant in certain specific contexts or subgroups.

### Limitations and Directions for Future Research

The present study has several limitations that should be considered when interpreting the findings. First, our assessment of the positive side of achievement goal theory may have limited our understanding of motivation in youth football. Specifically, we focused solely on the task-involving motivational climate and self-determined motivation, neglecting an examination of the ego-involving motivational climate and controlled motivation. To gain a more comprehensive understanding of motivation, future researchers should incorporate measures of ego-involving climate and controlled motivation, as they are also known to influence individuals' experiences and behaviors. Similarly, we solely measured enjoyment, and did not assess negative emotions (e.g., burnout or boredom). Negative emotions are important indicators of individuals' psychological well-being and can significantly impact their motivation and engagement. Thus, we may have missed opportunities to glean valuable insights regarding the complex interplay between positive and negative affective states in the youth football context. Another limitation of our study arises from our inability to collect data at the conclusion of the final season due to time constraints. Future investigators should explore a wider range of motivational factors influencing enjoyment or displeasure throughout an entire season (or even longer), collecting data at multiple time points. Furthermore, while our adoption of a short-term longitudinal design without an intervention allowed us to explore predictive relationships between the examined variables, it limited our ability to draw causal conclusions about the effects of some variables on others. Future researchers might incorporate intervention studies to investigate any causal effects of motivational factors on enjoyment and other outcomes in youth football. Implementing interventions that target and enhance task-involving climate and self-determined motivation might promote enjoyment and positive outcomes in young athletes. Also, our sample size was relatively small. Although our statistical analyses yielded significant results and our a priori power analysis suggested sufficient statistical power, a larger sample would have increased the generalizability of our findings. We merged multiple youth categories and both sexes into one global sample, potentially overlooking important age and sex related differences in motivational processes. Future researchers should include larger and more diverse samples, to allow for a more nuanced examination of motivational factors and their impact on enjoyment across different youth categories and sexes. Lastly, we did not include the measurement of competitive records, such as performance, goals, or ranking. While there is insufficient past research on this subject to conclusively establish whether objectively measured records would significantly impact the outcomes of our study (Noon et al., 2015), we also recognize this to be a potential limitation.

## Conclusions

Collectively, findings in this research highlight the significance of enjoyment, self-determined motivation, and a task-involving climate in understanding and promoting players’ enjoyment in youth football. Coaches, practitioners, and policymakers should prioritize strategies that enhance intrinsic motivation, provide opportunities for autonomy, and cultivate a supportive and growth-oriented environment. By doing so, they can maximize the enjoyment experienced by young football players and foster sustained engagement in the activity.
